# A Treatise on the Diseases and Physical Education of Children

**Published:** 1850-11

**Authors:** 


*A Treatise on the Diseases and Physical Education of Children.* By JOHN EBERLE, M. D., &c. &c. Fourth edition with cuts, and large additions, by THOMAS D. MITCHELL, M. D., &c. &c. Philadelphia, Lippincott & Grambo : 1850.

Professor Meigs has not been idle during the last few years, as his recent publications testify. Cognizant of the vast experience a large practice among children must have afforded him, we were rather disappointed in finding that he confined himself to "*some* of the diseases of children," and *in limine* we must beg leave to differ from the general custom of the day, to disclaim in the preface "any intention to make a systematic work on the subject, seeing that the place is already occupied with numerous valuable books, presenting a complete body of doctrines on children's diseases." Such an announcement cannot excuse any author, nor free him from the necessity of unfolding to his fellow laborers all the results which his abilities and opportunities have enabled him to attain.

The work before us is undoubtedly a valuable addition to the fund of information which has already been treasured up on the subjects in question. It is practical, and therefore eminently adapted to the general practitioner. We regret to find occasionally the same style pervading it, for which our esteemed author has hitherto been censured. He now and then takes liberties with his mother tongue, coining words where he chooses, and anastomosing them with those of other languages whenever it suits his fancy. We regret this careless style the more as he designs his publications chiefly for students; if he be desirous of impressing them with the importance and gravity of the subject he is treating, and with the utility of his instructions, let him recall the advice of the Roman satirist: "Si vis me flere primo tibi flendum est;" if he desire to secure the attention of his readers, he must treat his subject with the seriousness and dignity it demands.

Nor any the less objectionable are the theoretical flights in which our author now and then indulges, the more dangerous because they are so happily expressed.

In attaching a great deal of importance to the physiognomy of the sick child, we consider that Prof. Meigs is calling the attention of medical men to a point too often neglected, and from which they might derive great advantages in their prognosis of the diseases of children, thus confirming Mr. Jadelot's researches on this subject, to whom the profession is deeply indebted for information which had previously remained latent, but we cannot yield our implicit confidence to the author's views upon the voice of the child in crying, although we know that in this opinion he is also sustained by others. It has been beautifully said in reference to the undulatory theory of light, that the eye listens for light as the ear does for sound, but those who give the different aches a voice have certainly the advantage in originality.

Prof. Meigs is down stairs, hears a child cry, and by a species of mysterious knockings upon his own tympanum, instantly recognises from the peculiar tones of the child that it has the earache. He proves the correctness of his views by entering the room, and pressing upon the right ear; the infant only evinces surprise at his rudeness; he repeats the same movement upon the left, when lo! the same dulcet notes are poured forth hence it is the cry of the earache. Here is the key to all aches, so that every organ has its peculiar key, and when all become affected, all the stops are pulled out, a glorious diapason is formed, and we have a chorus of aches—"spectatum admissi risum teneatis amici." We prefer the views of Bouchut on this point, who in his recent work, "Sur les maladies des nouveaux nés," in the chapter headed "Du Cri" thus expresses himself: "Il n'y a guère qu' une maladie dans laquelle le cri présente des modifications importantes et caractéristiques, je veux parler du croup." Again, still further on, "la durée du cri des enfants n' indique pas autre chose qu'une douleur très vive, sans aucune rapport avec l'affection de tel ou tel autre organe," &c.

The first chapter is taken up with some very able remarks in reference to the diagnosis of the diseases of early infancy, the importance of which is forcibly dwelt upon; some of the diseases of uterine life, the non-viability of the child, the causes of children being born in an asphyxiated condition, and the treatment to be pursued under such circumstances, the formation of the navel, and lastly the retention of the meconium from inertia of the bowels or imperforate anus. Although these points are in the main well treated, still we regret their conciseness, and we would have wished our author to have drawn more upon his vast fund of experience and observation, and given us some chapters upon the physical training, and hygienic treatment of new born children.

We skip over the chapters on bloody tumors of the scalp, and inflammation of the eyes and mouth, as they require no particular remark.

Chapter 4th has for its subject Coryza. Dr. Meigs holds the opinion "that the nares are the orifices through which the vital air has access to the lungs, and that an instinctive sense teaches the new-born children to use them alone." He attributes the disease entirely to the nares becoming obstructed with a thick inspissated mucus; respiration being thus interfered with, the vitality of all the tissues becomes impaired from want of proper æration of the blood, the functions are interfered with, and emaciation takes place. The author has omitted to state that rapid emaciation very often ensues from the child's inability to suck, the mouth being constantly employed in keeping up the oxygenation of the blood; it is therefore requisite that the child should be fed with the mother's milk, by means of a spoon, since the fruitless efforts to suck aggravate its sufferings, and yet the want of nourishment, tending to augment the vitiation of the tissues, the child may die from inanition.

Dr. Meigs concludes his admirable chapter on this subject by recommending a constant lubrication of the nostrils with some animal oil to prevent the hardening of the mucus viscosities. He also dwells with much earnestness upon the treatment suggested by himself. We copy his own words. "In a very considerable number of cases. I have found the warmth of a flannel cap, worn upon the head, sufficient speedily to cure the malady, and I beg leave to assure the reader that, for the most part, little else is to be done, beyond giving very clear directions as to this method."—We have tried this *eureka*, and regret that we cannot confirm the author's views, having found that it has no advantages superior to those derived from keeping the child in a room of equal temperature, the disease in the majority of cases running its course.

We would call particular attention to the chapter on bowel complaints and early feeding, as containing most valuable suggestions.

It is unfortunately too common a practice to cram the newborn infant with gruel, or molasses and water, the physicians carelessly entrusting their delicate charges to the whims and freaks of officious nurses, whom neither time nor experience can teach, that in this, as in all similar circumstances, we should attend to the indications of nature. The peculiar secretion called colostrum, which issues from the mother's breast, is the repast which nature has destined for the child, and which from its very composition is peculiarly adapted to lubricating the internal surface of the digestive tube, to stimulate its contractions, to dilute the meconium, and consequently to facilitate its expulsion ; properties which our learned author does not appear to recognize, judging from the following remarks :—"I know not and believe it is not known, whether any peculiar function or power is connected with the early ingestion of the early milk of the mother, a fluid known to be characterised by the presence of a large quantity of colostrum."

The following chapters treat of infantile jaundice, and of the child's dress. We recommend the attentive perusal of the last named chapter to those practioners who as servile panderers to the morbid tendencies of the age would trammel the young and delicate infant by the bonds of fashion, regardless of the varieties of climate or the peculiar diathesis of the child.

We have now reached the chapter on Cyanosis Neonatorum, and in approaching this subject we would inform our readers, that we have reached our author's hobby-horse, and as, in the language of Sterne, " a man's hobby-horse is as tender a part as he has about him," we shall carefully endeavor to make no unprovoked strokes.

The first part of this chapter is taken up in considering the fœtal circulation; the author then proceeds to prove that, 1st, the nervous mass is the generator and conductor of biotic force, and 2dly, that oxygeniferous blood is indispensable to the evolution of life force. Professor Meigs' remarks on these points are fraught with information of the most practical kind, but we are at a loss to understand how he can consider them as consequent upon his own theory alone of the causes, inasmuch as they can be predicated of that of his opponents. Professor Meigs still maintains his former theory of this disease, viz., "its being entirely due to a persistent opening in the foramen ovale after birth, and an admixture of arterial and venous blood." He also persists in the treatment suggested by himself, of placing the child upon the right side and thus causing the valve of Botalli to close.

It seems to us that if the discoloration of the skin depends entirely upon the admixture of arterial and venous blood, we should always find in cases where discoloration existed the communication above named. Many cases, however, have been cited in which neither the foramen ovale nor the ventricular septum, was open, nor did there exist any other passage by which the arterial and venous blood could commingle, yet in all these there was the discoloration which is characteristic of cyanosis.

We could go on to prove, 1st, that cyanosis may exist without the admixture of blood; 2d, that there is no proportion between cyanosis and the degree to which the blood is mixed; and 3d, that complete mixture of the blood may take place without cyanosis. We might thus step by step sap the foundation of his whole theory of cyanosis. While upon this point, we would refer our readers to the excellent inaugural essay written on this subject by Moreton Stillé, M. D., of this city, and published in the American Journal of Medical Sciences for 1844, whose views have been entirely overlooked by Professor Meigs in all his writings on cyanosis; and yet Dr. Stillé examines the subject so ably and dispassionately, and presents such an array of facts to support his opinions, that we should have supposed they would have commanded the attention of so philosophical a mind as our author's. The result of the observations of Dr. Stillé prove, we think, most conclusively that congestion of the venous system, resulting from some obstruction in the right side of the heart, or in the pulmonary artery, impeding the return of the blood to the lungs, affords the best method of explaining cyanosis; and that if contraction of the pulmonary artery be taken as the type of all the lesions which may produce cyanosis, we are entitled to state, 1st, That it is present in every case of cyanosis. 2d. That it never exists without the concurrence of cyanosis. 3d. That it is an adequate explanation of the most important phenomena of the disease.

As for Dr. Meigs' proposed plan of treatment, and the great success which he says has attended it, we think that we cannot do better than copy the remarks of Mr. Norman Chevis upon this point, who, in an able article, published in the London Medical Gazette of March, 1847, after having announced that his own investigations are almost confirmatory of Dr. Stillé's inferences, uses the following language in respect to Dr. Meigs' plan of treatment. "Successful as this application of Dr. Meigs' theory may have proved, it is certain that his explanation of the fact is by no means demonstrative. So far from the patency of the foramen ovale being an essential concomitant of blue disease, it is well known that in a very considerable proportion of instances of cyanosis, the auricular septum is perfectly closed." . . . . . . Further on he adds, "The position of the body recommended by Dr. Meigs, is, however, well calculated to relieve the paroxysms from which subjects of congenital heart disease suffer, as it places nearly the whole of the voluntary muscles in a state of relaxation, thereby rendering the circulation through the extreme vessels as free as possible, and, (what is of more importance) as it facilitates the supply of arterial blood to the lungs, and to the brain." It is not, therefore, essential to the success of Dr. Meigs' treatment, that his views of the cause of the disease should be correct.

The chapter on Croup is an able exposition of the most recent views of the disease; and the great success which the author has had in this disease, should secure the greatest confidence in his statements.

The opinion that croup, though an inflammatory disease, is not without a very evident spasmodic element, is, we think, a most judicious one; and many practitioners in treating this disease shipwreck on this point, some entirely excluding the inflammatory, others the spasmodic. The following paragraph will, therefore, be read with interest:

"It is quite true that in spasmodic croup, or spasmodic laryngitis, it often happens that although the patient may have been suddenly attacked, and almost as suddenly relieved, he is notwithstanding left for some time after the relief, affected with signs of an altered condition of the windpipe; that is to say, he will be a little hoarse; and upon any attempts at rapid, or sudden aspiration, he will find that the croup sound is still there; as is the case also in the act of coughing, and this though, in any ordinary state of breathing, not the least sign of difficulty can be perceived. This seems to me to show, that what is called spasmodic croup, is not merely spasm, but there is a substratum of congestive, or inflammatory, disorder, which ought not to be lost sight of by the medical attendant," &c.

Dr. Meigs is a strong advocate for the lancet in this disease, and very justly observes, " a vast number of cases yield readily to a treatment without venesection. I advocate the precept to remember, always, that the lancet affords the surest guaranty of success and safety." Our own experience confirms the author's views, and we could relate numerous instances in which the attack was cut short by a prompt application of the lancet.

Dr. Meigs extols the use of the alum emetic, and we think with great propriety, for in our hands it has proved one of the most useful remedies in removing the inspissated mucus from the fauces and larynx, and at the same time it is so much more harmless than the domestic remedy, Coxe's hive syrup, a preparation, from the indiscriminate use of which, we have seen many untoward symptoms arise. We have for some time entertained the opinion that this was entirely too powerful a remedy to be entrusted in the hands of a mother or nurse, and administered at their discretion. It is undoubtedly accumulative in its effects, and we have the records of several cases in which inflammation of the gastro-intestinal mucous membrane supervened upon its use. We therefore deem it a duty to enter a caveat against our author's recommendation of it as a domestic remedy; the alum has in our hand's been quite as efficient.

The observations upon tracheotomy as an ultima ratio in croup, are sound and liberal, and contrast well with the croakings and anathemas of some of our medical men against this brilliant triumph of surgery.

We ourselves could add the authority of a recent case of tracheotomy performed by the same eminent surgeon, Prof. Pancoast, in which the result was as gratifying as that of young Repplier's; there is one point, however, in the treatment of tracheotomy which our author seems to have overlooked, and yet one we deem of vital interest, we refer to the introducing into the trachea, after the operation, a solution of nitrate of silver, sixty grs. to the ounce of water. This solution should be dropped by means of a quill used as a tâte liqueur, having found in our experience that the impetus with which the stream issues from the syringe, causes the trachea immediately to reject the fluid, whereas, by gradual instillation a great deal oozes down.

The chapter on pertussis is extremely interesting and practical; the origin of the disease is attributed entirely to a morbid condition of the medulla oblongata, unattended with any disease of the lungs or pharynx. The following is very much to the point:]

"I have already said that I look upon ordinary whooping cough, as a mere fit of coughing, which is peculiar not only by the long inspirations and the succession of incomplete expirations, and by the whoop, but also by the periodicity. And I repeat that if the patient have only the whooping cough, I generally leave him to nature for the cure, preferring to entrust him rather to her power than to the questionable conservatism of any therapeutics whatever."

The following remarks upon the condition of the stomach are of great utility.

"It is expedient in cases of pertussis to regulate the diet of the patient. An over rich and stimulating diet tends to develope the the gastric and circulatory complications of the malady in a positive manner," &c.

The chapter on laryingismus stridulus is in our opinion one of the most interesting in the book. The origin of this affection is seated by the author in the brain and diaphragm, especially the latter, and hence he proposes the name Phrenismus, as more properly indicating the real seat of the disease. Considering that the attacks by suspending the æration of the blood, vitiate the blood so as to render it either unfit for the nervous mass or even poisonous to it, Prof. Meigs very properly recommends us to cut short the attack, especially the primary attack, as rapidly as possible, "by applying a lump of ice wrapped in a handkerchief or napkin, to be applied to the epigastrium, and moved over the arch of the hypogastrium."

The author finishes his judicious treatment of the disease by the following recapitulations which on account of their soundness and usefulness we will give in full.

"In fine I believe our duty in the case consists—

1st. In making a correct diagnosis.

2d. In presenting proper explanations to the friends as to the prognosis, which is often unfavorable.

3d. In obviating the provoking causes, as swollen and distended gums, which are to be relieved by cutting them.

4th. In directions as to diet, dress, exposure, and all that concerns the hygiene of the case.

5th. In the use of counter-irritants and anti-spasmodics in the exhibition of tonics, and in attempts to defeat the demonstration of the attack by great quietude, and by applying cold to the region of the diaphragm; and lastly, in conducting the paroxysm, when formed, to the earliest and least mischievous possible conclusion, by the warm bath, and other measures."

We have now reached the last chapter in the book, in which the author treats of scarlet fever.

He first sets out with the announcement of his opinion "that scarlet fever is a non-contagious disease, whose cause is to be sought for in some intemperies of the air, or epidemic principle, of whose intimate nature I am wholly ignorant." The next question discussed is the nature of scarlet fever, which our author considers an inflammation of the true blood vessels or endangium, the inflammation being observable chiefly in the capillaries of the skin, mouth, throat and nostrils. In some cases the inflammatory affection seizes upon the capillaries of the brain, and more rarely upon those of the stomach and bowels. The author says:

"Seeing that the skin is an organ of vast extent, exceedingly vascular, and possessing important relations with the rest of the economy, we feel no surprise to observe the constitutional disorder produced by so extensive an inflammation as that of the whole derm. As the crasis of the blood depends upon the healthful force of the endangium, it is to be expected that extensive and violent disorders of the endangium shall produce great changes in the blood, and that these influences will exercise a pernicious influence throughout the economy. The nervous force, dependent as it is on the power of the blood to be charged with oxygen, fails under such conditions of the vital fluid, and the organs and functions in succession become overthrown."

Dr. Meigs considers that the different varieties of scarlet fever, result entirely from the peculiar set of capillaries affected: thus in scarlatina simplex, the capillaries of the skin are solely affected, in scarlatina anginosa those of the skin and of the mucous membrane of the fauces; in scarlatina maligna, the capillary endangium of the skin, fauces, Schneiderian membrane, tongue, parotids, heart, lungs, stomach or encephalon are all involved.

The author's pathology, however ingenious and interesting it may be, lacks certain links, without which his deductions are imperfect. In the first place it is by no means conceded that the endangium either makes the blood by transmitting to its elements the nervous force, or maintains the blood in a living state, by its presence and contact. 2d. Proofs are still wanting, that positive inflammation of any part of the lining membrane of the capillaries exists in this disease. The morbid appearances upon dissection of fatal cases of scarlet fever are by no means uniform, and Dr. Tweedie states that he has frequently been surprised on examining rapidly fatal cases, to find no morbid appearances that could explain the cause of death.

Dr. Meigs' remarks upon the treatment of the disease, are fraught with practical instruction. In treating the sick, says the author, we ought always conscientiously to avoid the administration of drugs and medicines, in the cases where we have no call for them." The practice of the present day, is unfortunately sadly at variance with this salutary caveat. "Melius anceps remedium quam nullum," is the cry which assails us on all sides. Masterly inactivty is as sedulously eschewed in the sick room as in the legislative halls, and the progressiveness of the age requires all the thunders of the materia medica to be hurled against the aggression of the disease, in whose ruins the mangled remains of the vis medicatrix naturœ may sometimes be discovered.

Dr. Meigs' experience is in favor of venesection in strong and plethoric subjects, where the fever is high. Affusion of the body with cold water is highly extolled by him, but we think that the sponging of the body is preferable to the method pointed out by the author. We were anxious to discover what views Dr. Meigs entertained, as regards the administration in this disease of any of the mercurial preparations. We have invariably found their employment attended with ill results, and have for some time sedulously avoided them. We should be glad to have the opinion of such experienced men as our author, upon this subject. Too much stress cannot be laid upon the author's instructions to keep the fauces and nostrils clear, not only in reference to the comfort of the child, but also with the view of preventing a vitiation of the blood from partial asphyxia.

We regret that our limits will not allow us to follow Professor Meigs any further, for although there is much in the pathology of this work which is fanciful and at variance with recent investigations, and we have not hesitated to designate those points in which we deemed the author to err, still we would remind our readers that stones are only thrown at those trees which are laden with fruit; the spots in the sun must be carefully mapped out and numbered, to be known; but the light and heat of the great luminary require no herald and no chronicler. Dr. Meigs' works have the same fascination which belongs to himself; his style is bold, fanciful and ingenious; he writes *curente calamo*, his thoughts trickling down from his brain to his pen; and though he sometimes forgets the sage precept, " sæpe stylum vertas," still, considering his constant occupation, and the immense toils which he undergoes, we are amazed that he can accomplish all that he does. Long may he be spared to grace the profession with his rare abilities.

We shall look forward for another volume from him, when, having retired from the toils and anxieties of one of the largest practices ever enjoyed by a medical man, in the shade of a mellow age, he can reveal to us more calmly, more dispassionately, and with more justice to himself, those mysteries which the labors of years have disclosed to him, years spent in unceasingly ministering to the sufferer, and in the practice of the most ennobling virtues.

We regret that the space occupied in noticing Professor Meigs' work, prevents us from dwelling at length upon the second which heads our remarks.

Dr. Mitchell's work is a reprint of Dr. Eberle's upon children, to which he has added a large appendix. We refrain from making any comments upon Dr. Eberle's part, inasmuch as it belongs to the past, and the profession have already pronounced judgment upon it.

We differ from Dr. Mitchell in our views, as regards the utility of bringing out another edition of Eberle, at a moment when we are flush in works in all languages upon the diseases of children, works combining in them all the experience of the past with the results of modern investigation. We admit that the importance of this branch of medical science demands the deepest attention, and exacts from us a most intimate acquaintance with every author that can shed any light upon so obscure a subject; and yet we think that the profusion which now exists is disadvantageous to the student and practitioner, placing them in the same category with the child cited by J. G. Rousseau, who, gathering shells by the seaside, begins at once by picking up an ample supply, and then desirous of securing all those which strike his eye as he moves onward, he alternately selects and rejects, until finally weary, overwhelmed by the number and hesitating which to choose, he ends by throwing all away, and returns home empty handed.

Dr. Mitchell's sequel contains a fair and concise exposition of some of the diseases of childhood. As we have not noticed anything original in the author's views, and finding that they are generally a fair summary of the opinions of the majority of writers upon the same points, we shall not pause to make any remarks. We would merely call particular attention to the chapters on "Infantile Mortality," and "Diseases acquired and inherited;" they are very interesting, and are entitled to serious consideration.

The author has a queer fancy for commingling Latin and English in his prescriptions, as we observed before in our notice of his work on Therapeutics; it would be far preferable to adopt one or the other. A hasty examination of Dr. Mitchell's work has led us to believe that it embodies all that is important and useful on the subject, and may therefore be safely consulted as a text book; but as it has not added materially to our previous information on the subject, we are forced to exclaim *cui bono!* If an author is not going to add to our previous store, if he cannot give us the results of his own daily observations and midnight watchings, in fine, if he have no original communication to offer, as we are strong in books, we beg "in mercy spare."

